# Supplementary material for: The Contribution of Multiplexing Single Cell RNA Sequencing in Acute Myeloid Leukemia
Source: Diseases. 2023 Jul 12;11(3):96. doi: 10.3390/diseases11030096 (PMC10366847; doi:10.3390/diseases11030096)
Supplement: Supplementary file 1 [file diseases-11-00096-s001.zip › diseases-2450513-supplementary.pdf]

Legend of supplementary data

Supplementary data S1. UMAP projection of PBMCs from AML patients grouped into different clusters, (1a - 1b) in UPN 23 and (1c-1d) in UPN 29. (1a) Grouping of PBMCs into twelve clusters (from 0 to 11) in UPN23SC, and into eleven clusters in UPN23SC-m (1b). (1c) Grouping of cells into sixteen different clusters in UPN29SC, compared to 13 clusters in UPN29SC-m (1d). Clusters are indicated with numbers, with percentages of cells in each mentioned cluster in parentheses.

Supplementary data S2. Marker genes per cluster in cells in UPN23 and UPN29. **(a)** Heat maps showing the most expressed genes in each cluster for UPN23 SC, **(b)** in UPN23 SC-m, **(c)** in UPN29 SC and **(d)** in UPN29 SC-m. Blue arrows indicate genes specific to LB, LT and NK cells (such as MS4A1, CD79A and B, NKG7, GZMA and GNLY) and red arrows show genes involved in the leukemogenesis process (MPO and SOX4).
