# Supplementary material for: The Contribution of Multiplexing Single Cell RNA Sequencing in Acute Myeloid Leukemia
Source: Diseases. 2023 Jul 12;11(3):96. doi: 10.3390/diseases11030096 (PMC10366847; doi:10.3390/diseases11030096)
Supplement: Supplementary file 1 [file diseases-11-00096-s001.zip › Supplementary data.pptx]

## Slide 1
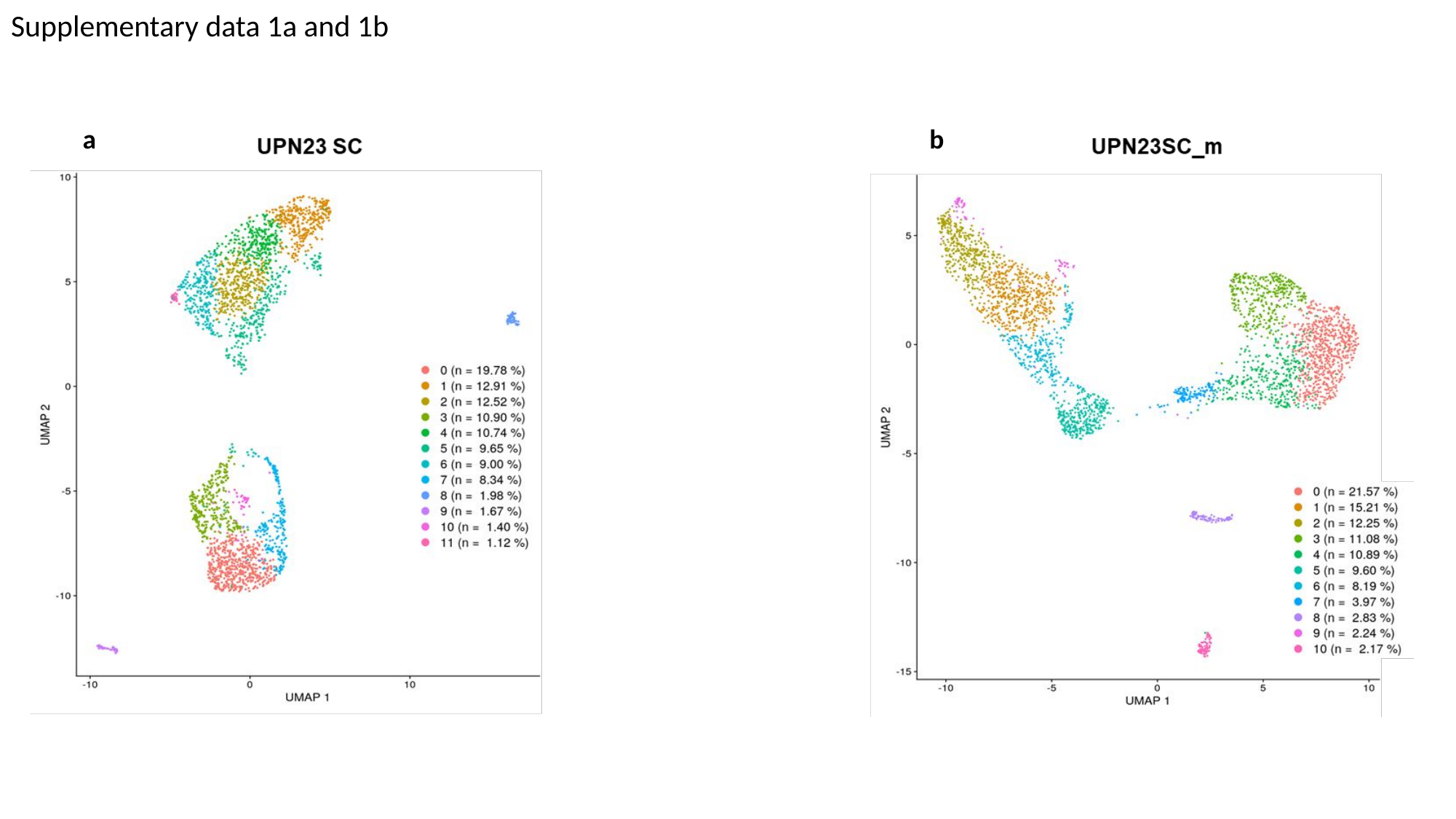

Supplementary data 1a and 1b
a
b

## Slide 2
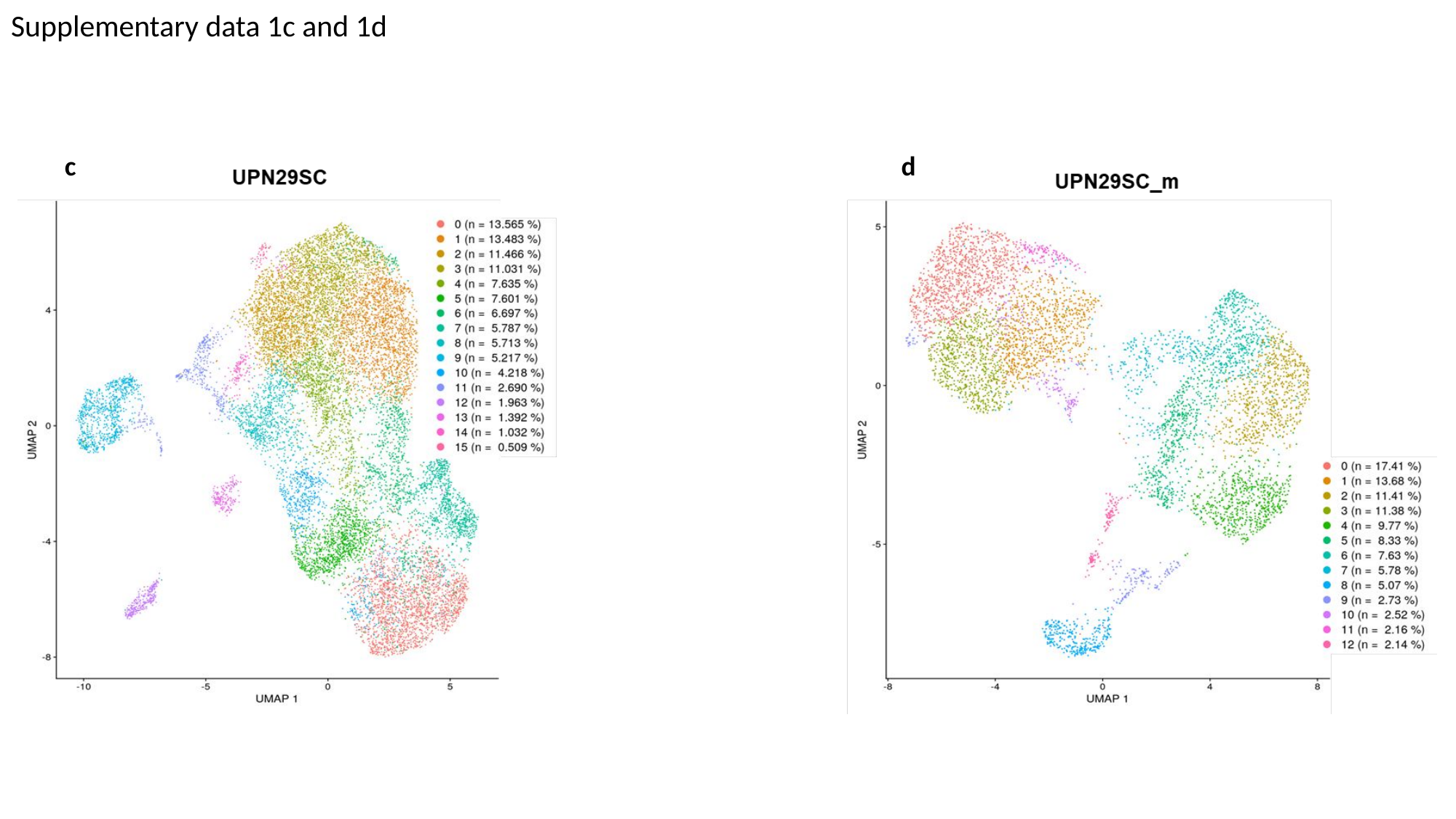

Supplementary data 1c and 1d
c
d

## Slide 3
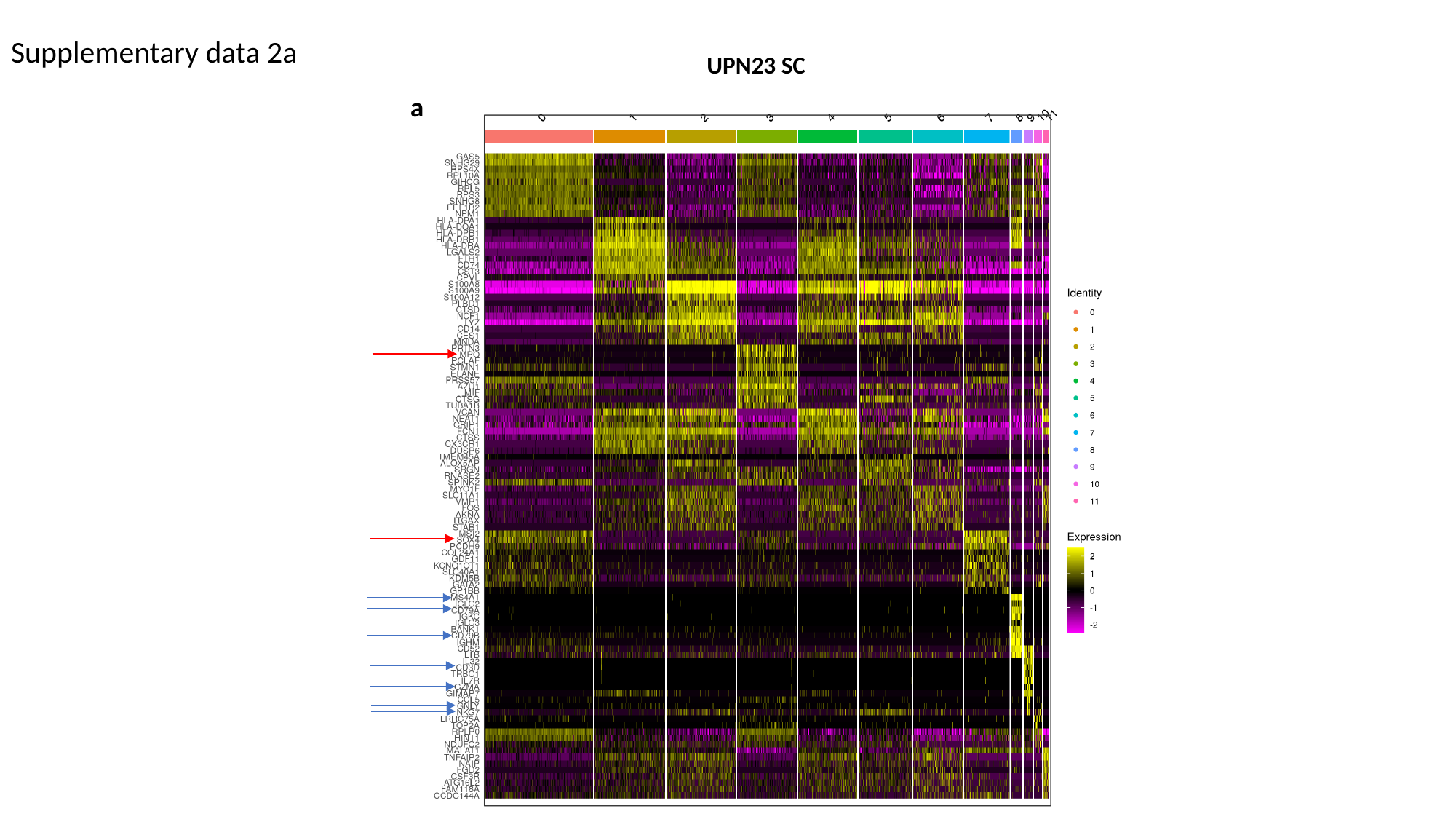

# Supplementary data 2a
UPN23 SC
a

## Slide 4
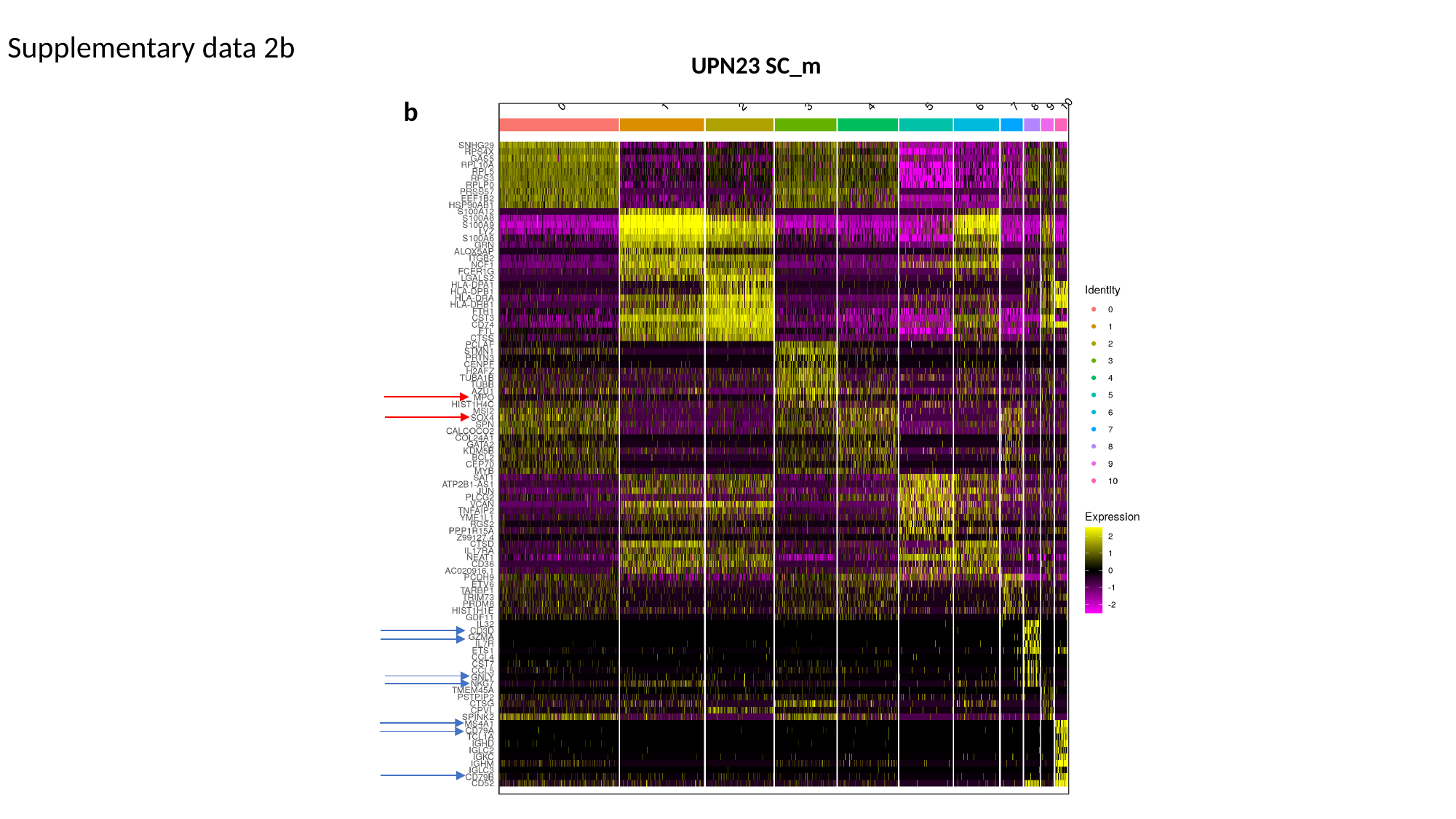

# Supplementary data 2b
UPN23 SC_m
b

## Slide 5
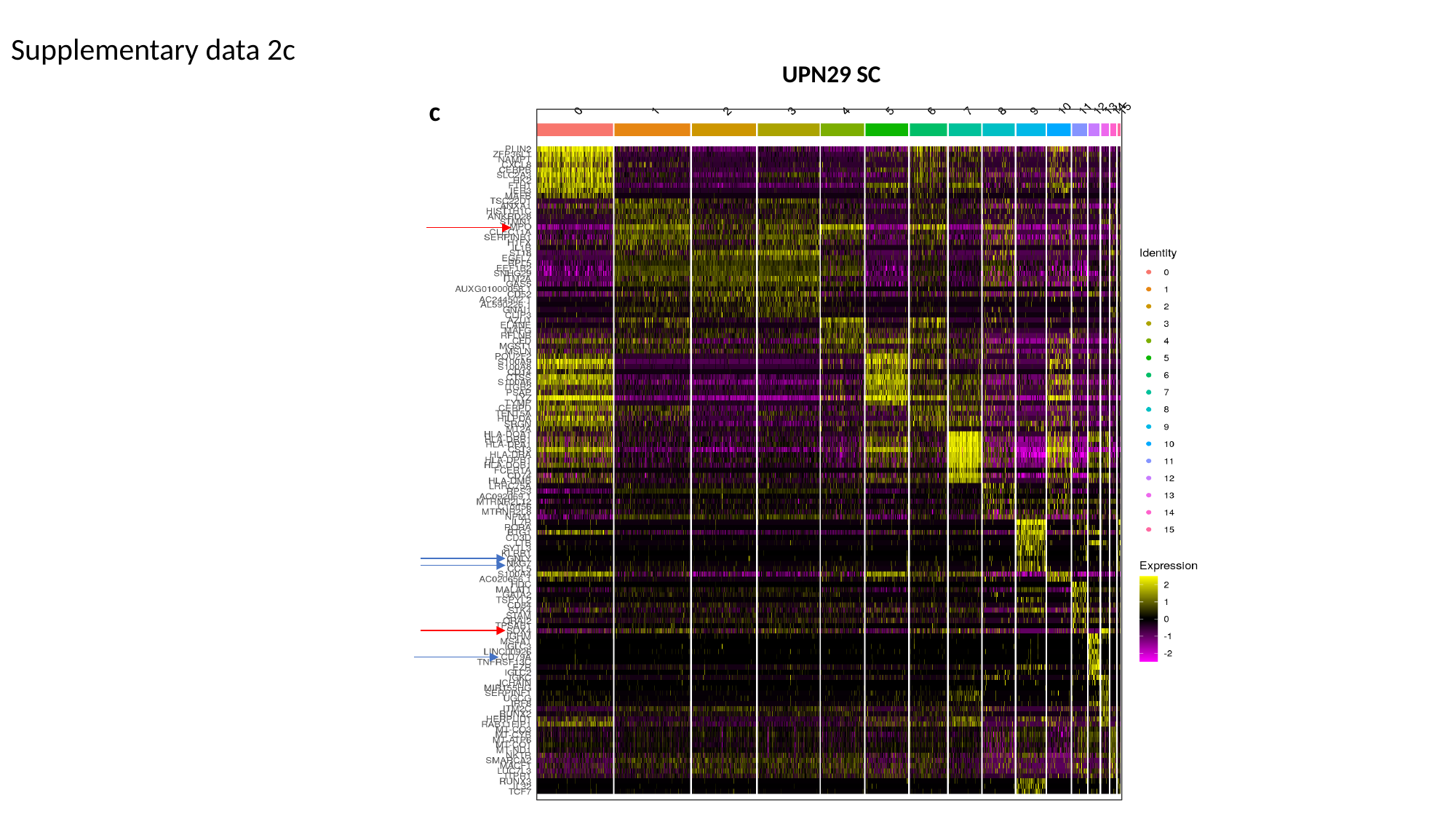

# Supplementary data 2c
UPN29 SC
c

## Slide 6
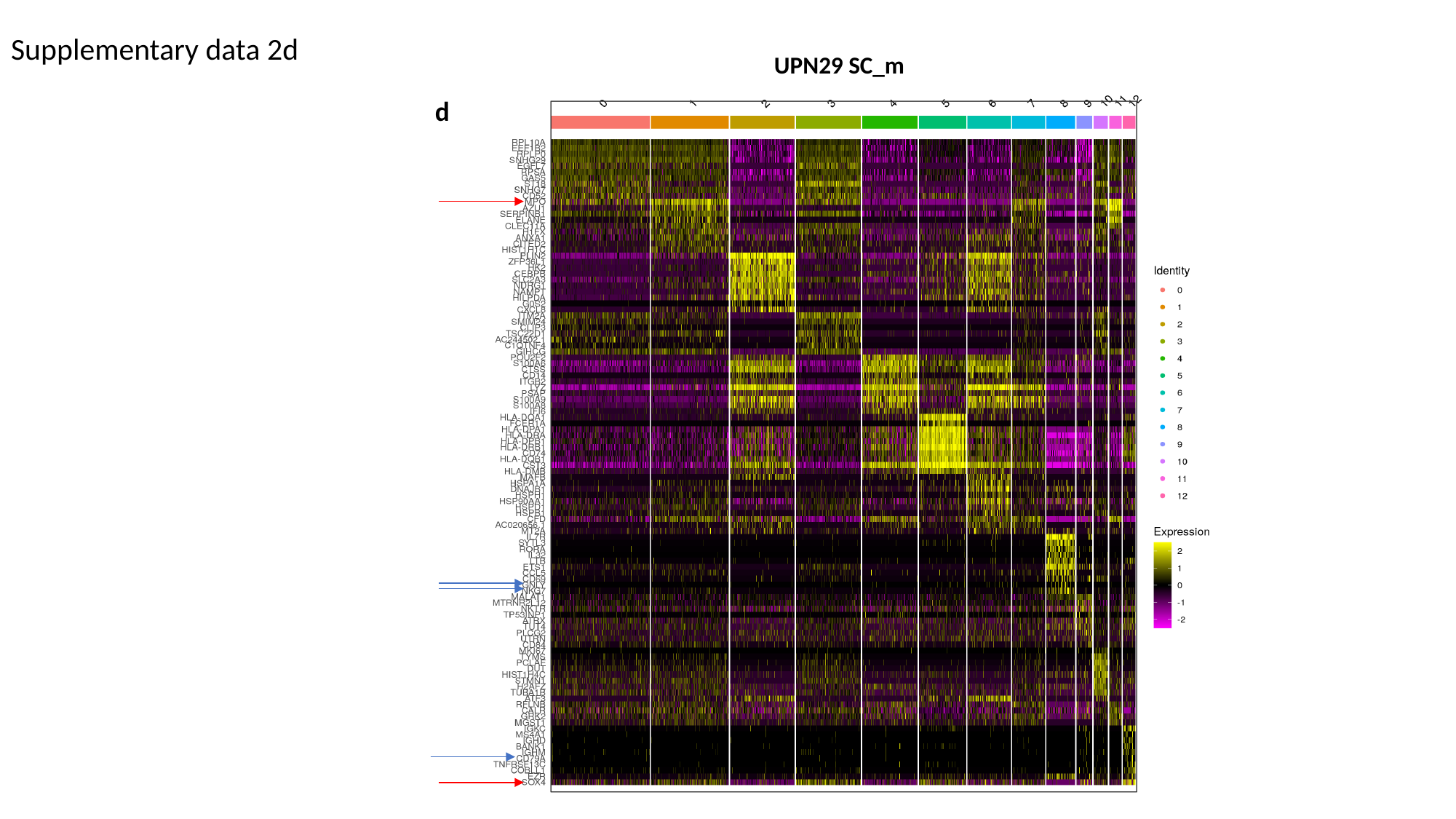

# Supplementary data 2d
UPN29 SC_m
d
